# Carbonyls and Carbon Monoxide Emissions from Electronic Cigarettes Affected by Device Type and Use Patterns

**DOI:** 10.3390/ijerph17082767

**Published:** 2020-04-17

**Authors:** Yeongkwon Son, Chiranjivi Bhattarai, Vera Samburova, Andrey Khlystov

**Affiliations:** Division of Atmospheric Sciences, Desert Research Institute, Reno, NV 89512, USA; yeongkwon.son@dri.edu (Y.S.); chiranjivi.bhattarai@dri.edu (C.B.); vera.samburova@dri.edu (V.S.)

**Keywords:** electronic cigarette, vaping topography, carbonyls, carbon monoxide, nicotine

## Abstract

Dangerous levels of harmful chemicals in electronic cigarette (e-cigarette) aerosols were reported by several studies, but variability in e-cigarette design and use patterns, and a rapid development of new devices, such as JUUL, hamper efforts to develop standardized testing protocols and understand health risks associated with e-cigarette use. In this study, we investigated the relative importance of e-cigarette design, power output, liquid composition, puff topography on e-cigarette emissions of carbonyl compounds, carbon monoxide (CO), and nicotine. Four popular e-cigarette devices representing the most common e-cigarette types (e.g., cig-a-like, top-coil, ‘mod’, and ‘pod’) were tested. Under the tested vaping conditions, a top-coil device generated the highest amounts of formaldehyde and CO. A ‘pod’ type device (i.e., JUUL) emitted the highest amounts of nicotine, while generating the lowest levels of carbonyl and CO as compared to other tested e-cigarettes. Emissions increased nearly linearly with puff duration, while puff flow had a relatively small effect. Flavored e-liquids generated more carbonyls and CO than unflavored liquids. Carbonyl concentrations and CO in e-cigarette aerosols were found to be well correlated. While e-cigarettes emitted generally less CO and carbonyls than conventional cigarettes, daily carbonyl exposures from e-cigarette use could still exceed acute exposure limits, with the top-coil device potentially posing more harm than conventional cigarettes.

## 1. Introduction

Electronic cigarettes (e-cigarette) have been assumed to pose less harm than conventional cigarette products, but e-cigarette vaping is not risk free [1]. Indeed, e-cigarette is a source of exposures to various air toxics. Previous studies demonstrated that e-cigarette aerosols contain carbonyls [2,3,4,5,6,7,8], volatile organic compounds [9,10], and metals [11,12]. Carbonyl compounds have been receiving a lot of attention so far as they were found to be the most abundant toxic species in e-cigarette aerosols [7].

E-cigarette carbonyl emissions were reported to vary with e-cigarette device construction, power output, and e-cigarette liquid (e-liquid) composition (e.g., base material, flavorings) [3,4,5]. For a given e-cigarette, a higher power output, and thus higher e-cigarette coil temperature, results in higher carbonyl emissions [4,5,6,8,13]. The e-cigarette construction (e.g., top vs. bottom coil, power per coil surface area, etc.) also influences carbonyl emissions as it affects e-liquid supply to the wick [3,5] and coil temperature distribution [14]. E-liquid composition, especially the flavoring compounds, is another significant factor that affects e-cigarette carbonyl emissions [3,15]. Very little is known about the effect of puff topography, such as puff duration and puff flow rate, on e-cigarette emissions. Puff flow rate was reported to affect coil temperature and thus e-liquid evaporation rate [16]. E-cigarette nicotine yields were shown to increase with puff duration, while puff flow rate had no effect [14]. E-liquid consumption and emissions of some carbonyls were observed to depend on puff flow rate [17].

Differences in device types and testing protocols (e.g., e-cigarette power, e-liquid composition, and puff parameters) used in previous studies [4,5,8] make it difficult to inter-compare results and draw conclusions about the relative safety of particular e-cigarette devices and e-liquids. If puff topography has a strong effect on e-cigarette emissions, translating results of laboratory studies performed using fixed puff parameters to user exposure becomes problematic because puff topography among e-cigarette users varies widely. Puff durations employed by users of the same e-cigarette device were reported to vary from 0.6 s to over 10 s [18]. In a study of 23 daily e-cigarette users, e-cigarette power output was reported to vary between 5 and 60 W, puff volume varied between 10–251 mL, and puff duration between 1.3 and 5.8 s [19]. Understanding of the relative importance of different parameters affecting e-cigarette emissions is needed for formulating standardized testing protocols and translating laboratory results to real-life exposure.

Carbon monoxide (CO) emissions from e-cigarette have been understudied [20,21], because a few studies showed that exhaled CO concentrations among e-cigarette users were significantly lower than conventional tobacco smokers [7,22,23,24]. However, CO can be formed in e-cigarettes through thermal decomposition of e-liquid base materials (propylene glycol (PG) and vegetable glycerin (VG)) [25] and some flavoring compounds [26]. Therefore, CO could serve as a useful indicator to study chemical processes occurring in e-cigarette.

In this study, we investigated the impact of e-cigarette construction, power, puff topography, and e-liquid composition on carbonyl, CO, and nicotine emissions. We tested four popular e-cigarette devices, each representing one of the popular e-cigarette construction types: A cig-a-like, a top-coil, a ‘mod’ with a sub-ohm coil, and a ‘pod’ system (i.e., JUUL). In 2014, the e-cigarette market in the U.S. was $2.5 billion (40% was for cig-a-like and 60% was for tank-type, ‘mod’, and other type devices). Currently, the ‘pod’ type e-cigarettes including JUUL are the most popular e-cigarette device in the rapidly increasing U.S. e-cigarette market, especially among teenagers [27], capturing over 72.1% of the e-cigarette market [28]. Very little is known about chemical composition of aerosols produced by the pod devices [9,29].

## 2. Materials and Methods

### 2.1. E-Cigarette Devices and E-Liquids

Table 1 lists e-cigarette brands, e-liquid types, and vaping conditions used in this study (more details can be found in Figure S1 and Table S1). Four e-cigarette devices were tested: A cig-a-like device with a single coil atomizer (V2, VMR products, LLC, Miami, FL, USA), a single-top-coil e-cigarette (eGo CE4, Shenzhen Somple Technology, Shenzhen, China), an adjustable power ReuLeaux RX200 ‘mod’ (WISMEC Electronics, Guangdong, China) with an Aspire Cleito atomizer (Shenzhen Eigate Technology, Shenzhen, China) equipped with a replaceable Clapton-style 0.4 Ω Kanthal coil, and a JUUL ‘pod’ system (PAX Labs, San Francisco, CA, USA). These devices represent a progression of e-cigarette device designs with the JUUL being currently the most popular device in the USA [27]. Since e-liquid PG:VG ratio and, especially, e-liquid flavor were shown to affect e-cigarette emissions [3,6], several e-liquids were tested in this study. Tierney et al. [30] have shown that flavoring chemicals differ among “flavor classes”. For example, sweet and fruit-like e-liquids contain mainly esters and alcohols, while cream-like flavored e-liquids contain carbonyls. Thus, e-liquids representing tobacco, fruit, cream, and mint flavor families were tested in this study (Table 1).

### 2.2. E-Cigarette Aerosol Generation

E-cigarette aerosols were generated using a lab-made automated vaping system (Figure S2). The effect of puff topography was tested by controlling either puff duration or puff flow rate (Table 1). The puffing parameters used in this study cover the range of e-cigarette vaping topography data reported in the literature (80.4–133 mL volume and 3.3–4.16 s duration) [18,19,31,32,33]. A square-shaped flow profile was used as it was observed during actual e-cigarette use [19,34]. A 30-s puff interval was used throughout the study. The effect of power output was investigated using the ‘mod’ device, because it was the only device used in this study that allowed adjusting power output.

### 2.3. CO Measurements

Concentrations of CO emitted from the e-cigarettes were measured using a Model 8830 CO Analyzer (Teledyne Monitor Labs, Englewood, CO, USA) connected to the vaping machine via a 2-m-long, ¼-inch outer-diameter Teflon tubing (Figure S2). A long piece of tubing was used to equilibrate the sample air temperature to the room temperature (24 ± 1 °C). A quartz fiber filter was installed at the inlet of the CO analyzer to protect the instrument. The analog output of the analyzer was continuously recorded at a rate of 5 Hz using a digital-analog converter (LabJack U6 series, LabJack Corporation, CO, USA). The CO analyzer was calibrated daily using a mixture of zero air and a CO standard gas (Airgas, Radnor, PA, USA). The total amount of CO produced per puff was calculated by integrating peaks in CO readings and multiplying them by the instrument sample flow rate (Figure S3). No interference from PG or VG in CO measurements was detected.

### 2.4. Carbonyl Analysis

The 2,4-Dinitrophenylhydrazine (DNPH)-coated glass fiber filters (ORBO 827, Supelco, Folsom, CA, USA) followed by DNPH cartridges (Sep-Pak XPoSure Plus Short Cartridge, WAT047205, Waters, Milford, MA, USA) were used to collect carbonyl compounds generated by five e-cigarette puffs. All measurements were done in triplicate. A combination of DNPH filters with DNPH cartridges was used to ensure that both particle- and gas-phase carbonyls were collected. After collection, filters and cartridges were extracted with 2 mL of acetonitrile (HPLC grade, EMD Millipore, Burlington, MA, USA). A 2-μL aliquot was then injected into an HPLC system (Waters 2690 Alliance with a model 996 photodiode array detector, Waters, Milford, MA, USA) equipped with a Polaris 3 column (C18-A, 3 μm, 100 × 2.0 mm, Agilent Technologies, Santa clara, CA, USA). DNPH-carbonyl adducts were quantified at 360 nm, while full spectrum readings (210–400 nm) were used to confirm the identity of individual compounds. Carbonyl concentrations were quantified using six-point external calibration curves prepared from a certified calibration mixture (AccuStandard, New Haven, CT, USA). Details of the analytical method and limits of detection can be found in Tables S2 and S3.

### 2.5. Nicotine Analysis

To determine the amount of nicotine produced by the e-cigarette devices, five puffs of e-cigarette aerosols were collected on a glass fiber filter (MilliporeSigma, Burlington, MA, USA). Filters were then spiked with 40 μg of quinoline (98%, Sigma-Aldrich, Saint Louis, MO, USA) and extracted with 4 mL HPLC grade methanol (EMD Millipore, Billerica, MA, USA). Quinoline was used as an internal standard due to its chemical similarity to nicotine, no interference in nicotine analysis, and its absence in the samples. Filter extracts were analyzed using the HPLC system described above (Waters 2690 equipped with a Polaris 3 column). External standards of nicotine (≥99%, Sigma-Aldrich, Saint Louis, MO, USA) and quinoline were prepared and quantified at 260 and 220 nm wavelengths, respectively. It should be noted that JUUL aerosols contain nicotine salts. Since these salts have a similar UV absorption maximum at 255–260 nm [35], they were quantified using the same nicotine standard. Details of nicotine analysis can be found in Tables S4 and S5.

### 2.6. Statistical Analyses

All statistical analyses in this study were done using the R software package version 3.4.3 (R Development Core Team, Vienna, Austria). Two-tailed Student’s t-tests and simple linear regression analysis were used to assess the effects of different parameters tested in this study. Pearson’s correlation was used to assess relationship between CO and carbonyl compound emissions. Significances were determined at *p* = 0.05.

## 3. Results and Discussion

### 3.1. Carbonyls and CO Emissions from the Different E-Cigarettes

**Figure 1:**
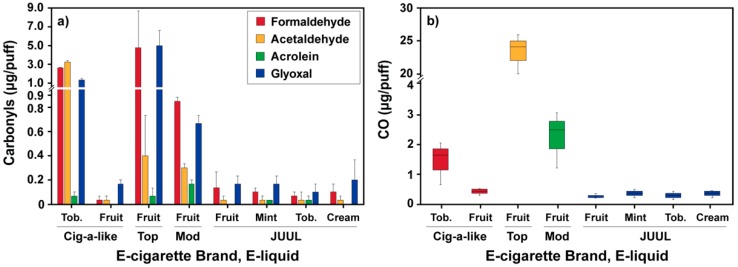
(**a**) Carbonyl and (**b**) CO concentrations for the combinations of the four different e-cigarette brands (cig-a-like, top-coil, ‘mod’, and JUUL) and flavored e-liquids (tobacco, fruit, mint, and cream flavored). The 50-watt power output was used for ‘mod’ device. Vaping topography was 100-mL puff volume, 4-s puff duration, and 30-s puff interval for all conditions (n = 3).

Carbonyl emissions varied among the e-cigarette devices and different e-liquids types (Figure 1a). Our carbonyl emission result was similar to earlier studies that reported higher carbonyl emissions from top-coil devices than other e-cigarette construction types (e.g., cig-a-like and bottom coil) [3,4,5,6]. The cig-a-like e-cigarette device with a tobacco-flavored e-liquid generated significantly higher formaldehyde (2.65 ± 0.06 μg/puff), acetaldehyde (3.27 ± 0.17 μg/puff), and glyoxal (1.35 ± 0.17 μg/puff) than the same e-cigarette with a grape-flavored e-liquid (*p* < 0.001). Notably, acetaldehyde emission from the cig-a-like e-cigarette with the tobacco-flavored e-liquid was significantly higher than all the other e-cigarettes (*p* < 0.001). The top-coil device generated the highest concentrations of formaldehyde (4.80 ± 3.88 μg/puff) and glyoxal (5.01 ± 1.65 μg/puff) among the tested e-cigarettes. The ‘mod’ device formed 0.17 ± 0.03 μg of acrolein per puff which was 3 to 6 times more than the acrolein concentrations emitted from the other e-cigarettes (*p* = 0.048). The cig-a-like e-cigarette with the grape-flavored e-liquid had the lowest carbonyl emissions followed by the JUUL. Among the JUUL flavors, fruit-flavor generated the highest amounts of formaldehyde (0.14 ± 0.04 μg/puff, *p* = 0.074) than the other flavors (0.07–0.10 μg formaldehyde/puff).

Figure 1b shows CO emissions for the four e-cigarette devices. CO emissions of the cig-a-like device with the tobacco-flavored e-liquid were 1.4 ± 0.7 μg/puff, which is 3.5 times higher than those produced by the same e-cigarette with the grape-flavored e-liquid (0.4 ± 0.1 μg/puff, *p* = 0.070). The top-coil device generated significantly larger amounts of CO (23.3 ± 3.1 μg/puff) than any of the tested devices (*p* < 0.001). The cig-a-like and the JUUL with different e-liquids generated 16-86 times less CO (0.4 ± 0.1 μg/puff) than the top-coil device. The ‘mod’ e-cigarette generated 10 times less CO (2.2 ± 0.9 μg/puff) than the top-coil device even though both devices were filled with the same strawberry-watermelon flavored e-liquid. To our knowledge, there was no systematic study on the direct e-cigarette CO emissions to date. Our results demonstrate that e-cigarettes generate CO along with carbonyls. Similarly to carbonyls, CO is likely emitted due to thermal decomposition of e-liquid components [25,26]. Further in the paper, we will provide more evidence that this is indeed the case.

### 3.2. Effect of Vaping Topography on E-Cigarette Emissions

**Figure 2:**
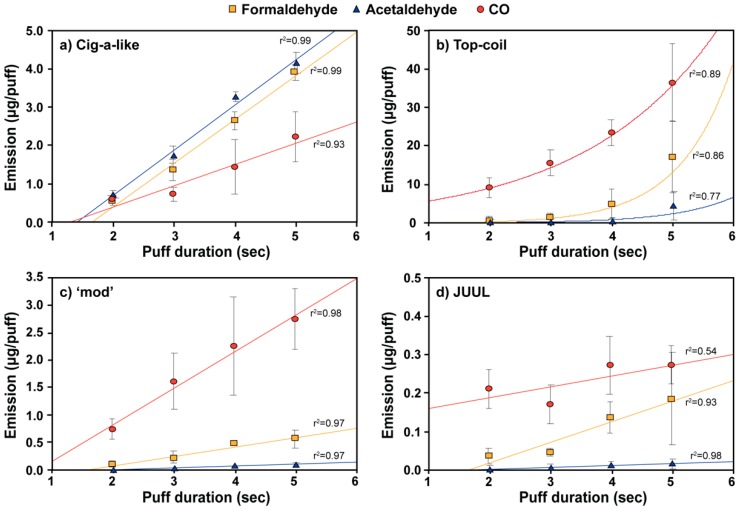
Dependence of carbonyl and CO emissions on puff duration for the (**a**) cig-a-like, (**b**) top-coil, (**c**) ‘mod’, and (**d**) JUUL ‘pod’ e-cigarette devices at 25 mL/s puff flow rate (error bars represent standard deviation of three independent measurements). The 50-watt power output was used for ‘mod’ device. Linear and, in the case of the top-coil device, exponential regression lines for each compound are also shown.

Figure 2 presents formaldehyde, acetaldehyde, and CO emissions for the four tested e-cigarettes under different puff durations. Detailed carbonyl concentrations and linear regression parameters (nonlinear regression parameters for the top-coil e-cigarette) under different puff durations are tabulated in Tables S6–S8. CO and carbonyl emissions for cig-a-like, ‘mod’, and JULL showed statistically significant increases with the puff duration (*p* = 0.007), except for JUUL emissions of CO (*p* = 0.100). The amount of heat transferred to the coil was directly proportional to the puff duration. This explains the linear relationships between the puff duration and the pollutant emissions observed in our study. A similar effect of puff duration on nicotine e-cigarette yields have been reported elsewhere [14]. The intercepts of regression lines with the x-axis indicate a minimum puff duration at which measurable carbonyl and CO emissions occur. Based on the estimated regression parameters, the tested e-cigarette devices start generating toxic compounds after approximately 1 s since e-cigarette devices need some time to heat up the atomizer coils to reach certain stable temperatures [4].

It should be noted that the top-coil e-cigarette emissions showed an exponential increase with puff duration (Figure 2b). Even though all top-coil emissions could be approximated with a linear relationship due to the large variability in the data, a nonlinear (exponential) regression showed a better fit with lower residual errors than linear regression. For CO and formaldehyde emitted from the top-coil device, the exponential dependence was statistically significant (*p* = 0.027), with acetaldehyde and acrolein demonstrating a weaker significance (*p* = 0.274 and *p* = 0.055, respectively). The significant nonlinear increase of carbonyl and CO emissions from the top-coil device with puff duration could be due to limited liquid transfer capacity of the wick in the top-coil devices. An imbalance in heat vs. e-liquid transfer could lead to overheating of the e-liquid increasing generation of carbonyl and CO.

**Figure 3:**
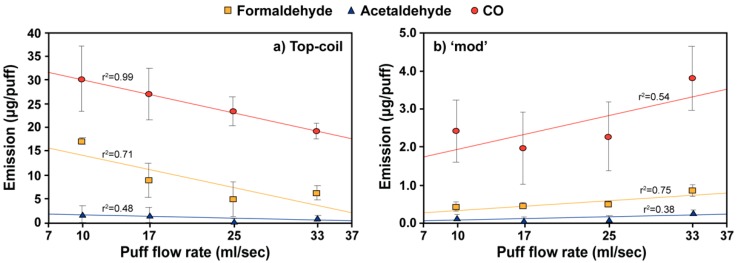
Impact of puff flow rate on carbonyl and CO emissions for the (**a**) top-coil and (**b**) ‘mod’ e-cigarette devices for a 4-s puff duration. The tested flow rates of 10, 17, 25, and 33 mL/s correspond to 40, 67, 100, and 133 mL puff volumes. A 50-watt power output was used for ‘mod’ device. Error bars represent standard deviation of three independent measurements. Regression lines for each compound are also shown.

Carbonyl and CO emissions for the top-coil and ‘mod’ e-cigarette devices were found to be fairly insensitive to the puff volume (Figure 3, Tables S9 and S10). For the top-coil e-cigarette, CO and all carbonyls but acetaldehyde emissions significantly decreased with increasing puff flow rates (*p* = 0.047). Acetaldehyde emission from the top-coil e-cigarette also showed a decreasing trend, though with a weak statistical significance (*p* = 0.266). In contrast, the ‘mod’ device generated higher carbonyls and CO at higher flow rates but only formaldehyde showed a significant positive slope (*p* = 0.003). The cig-a-like and JUUL devices could not be tested at puff flow rates below 17 mL/s because such flows could not activate operation of these devices. By comparing estimated regression parameters (Table S10), higher puff flow rates did not change carbonyl emissions for the cig-a-like and JUUL e-cigarettes (*p* > 0.052) except for glyoxal from the cig-a-like device (*p* = 0.002).

The dependence of carbonyl and CO emissions on the puff flow rate appears to vary with the e-cigarette construction. In our study, e-cigarette carbonyl and CO emissions showed a decreasing trend with increasing puff flow rate for the top-coil device, while the ‘mod’ e-cigarette showed an opposite, though weak, trend. Our results with the top-coil e-cigarette were consistent with previous reports. Previous studies showed decreased total particle mass concentration [36] and carbonyl emissions [17] at higher flow rates using cig-a-like, top-coil, bottom-coil, and JUUL devices. Faster air flows through the heating chamber were shown to decrease coil temperature [16], leading to lower aerosol and carbonyl emissions. However, in our study, the ‘mod’ e-cigarette generated more carbonyls and CO at higher flow rates. Different emission trends for the top-coil and ‘mod’ device could be due to differences in e-cigarette construction and properties (e.g., power output, chamber design, air hole size, air flow regime, coil resistance, etc.), though the exact cause of this was unclear and needs to be further studied.

These findings demonstrate that the effect of vaping topography should be carefully considered to test e-cigarette chemical emissions. Previous reports used puff durations of 1 to 2 s [6,37,38,39,40] and vaping flow rates of 10–16.7 mL/s [4,5,8,17,41,42] to measure the potentially harmful emissions from e-cigarettes. Depending on the e-cigarette construction, levels of potentially harmful compounds reported in the previous studies can cause the over- or under-estimation of the actual exposures since e-cigarette users showed much longer puff durations (3.5 to 4 s) and higher puff flow rates (24–38 mL/s) [31,32].

### 3.3. Effect of Power Output and Flavor on CO Emission

**Figure 4:**
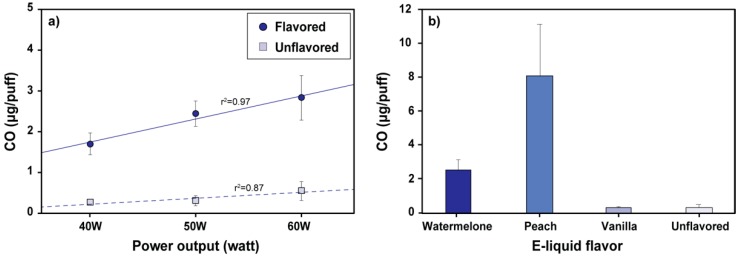
Effect of (**a**) e-cigarette power output and (**b**) e-liquid flavor on CO emissions. Power output effects were tested using 40 mL, 4-s puffs with a 30-s inter-puff interval with strawberry-watermelon flavored and unflavored e-liquids. Emissions of different flavor e-liquids were tested with the same puff parameters at 50-watt power output. Error bars represent standard deviation of three independent measurements.

Figure 4 shows CO emissions measured at different power outputs and using different e-liquid flavors with the ‘mod’ e-cigarette. A clear, linear increase of CO emissions with e-cigarette power output was observed for the strawberry-watermelon flavored e-liquid (Figure 4a). CO emissions from an unflavored liquid (PG:VG = 3:7) were significantly lower than those from the flavored liquid at all tested power outputs (Figure 4b). CO emissions increased by 0.057 μg/puff per 1 W of power for the flavored e-liquid (*p* < 0.001) and by 0.014 μg/puff per 1 W for the unflavored e-liquid (*p* < 0.001). Flavored e-liquids generated significantly higher (5- to 7-fold) CO than unflavored e-liquids under the same power output (*p* < 0.001). Strawberry-watermelon and peach-lemonade flavored e-liquids generated 2.5 ± 0.6 μg/puff and 8.1 ± 3.1 μg/puff of CO, respectively, significantly more than the vanilla-almond milk flavored (0.4 ± 0.1 μg/puff, *p* < 0.001) and unflavored (0.3 ± 0.1 μg/puff, *p* < 0.001) e-liquids (Figure 4b).

Increasing carbonyl emissions with increasing e-cigarette power is a well-known phenomenon: Applying a higher power to the coil increases its temperature, facilitating thermal decomposition of e-liquid components [4,5,6]. The co-occurrence of CO and carbonyl emissions in our experiments and the fact that CO emissions increase with e-cigarette power output indicate that, similarly to carbonyls, CO is produced by thermal decomposition of e-liquid constituents [20,21]. The observed variation in CO and carbonyl emissions among different flavored and unflavored liquids confirms the fact that these pollutants originate due to e-liquid breakdown and not because of decomposition of a liquid-starved wick material (the so-called dry puff phenomenon). Flavoring compounds in e-liquids were shown to be thermally unstable at conditions encountered in e-cigarettes, being responsible for the major part of carbonyls emitted by e-cigarettes [3]. Our results show that, similarly to carbonyl emissions, CO production in e-cigarettes appears to be dominated by flavoring compounds. The observed differences in CO emissions between flavored e-liquids are likely due to differences in thermal stability of individual flavoring compounds or differences in thermal decomposition pathways leading to CO formation. Further studies on the thermal breakdown of flavoring chemicals are desirable for better understanding of e-cigarette CO and carbonyl emissions.

### 3.4. Correlation between Carbonyls and CO Emissions

**Figure 5:**
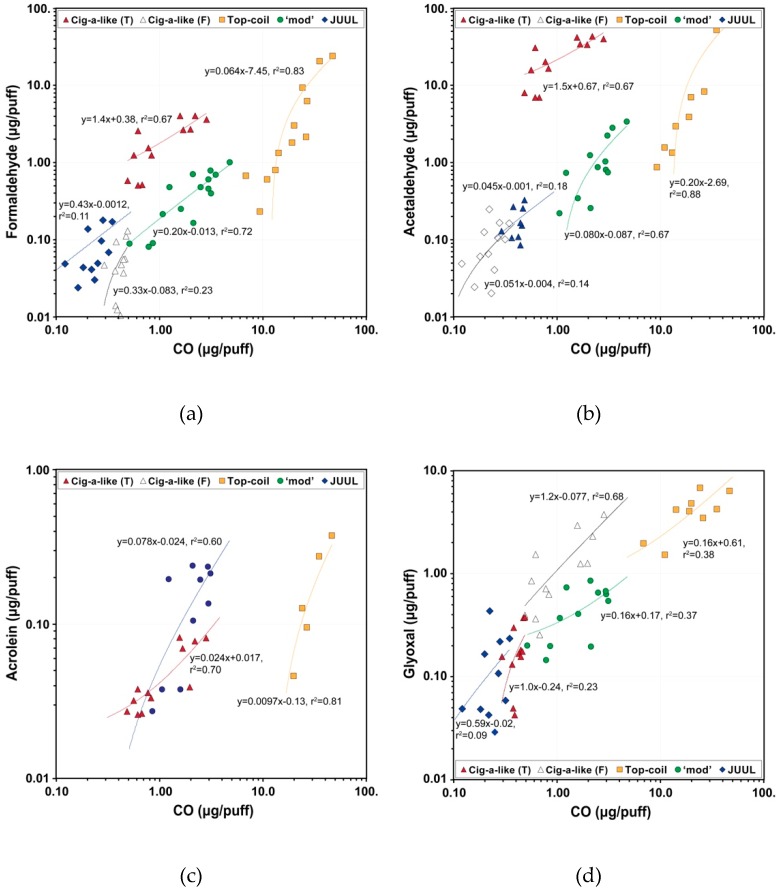
Relationship between CO and (**a**) formaldehyde, (**b**) acetaldehyde, (**c**) acrolein, and (**d**) glyoxal in e-cigarette aerosols. Cig-a-like e-cigarette was tested using tobacco (T) and grape (F) flavored e-liquids. Fruit flavored e-liquids were used for top-coil, ‘mod’, and JUUL e-cigarettes.

Figure 5 and Table 2 show a relationship and Pearson’s correlation coefficients, respectively, between CO and major carbonyls in e-cigarette aerosols, which were measured simultaneously. Formaldehydes emitted from the top-coil device (*r* = 0.909, r^2^ = 0.83), ‘mod’ e-cigarette (*r* = 0.847, r^2^ = 0.72), and cig-a-like device with tobacco flavored e-liquid (*r* = 0.820, r^2^ = 0.67) were significantly correlated with CO levels. Measured CO emissions also correlated well with other carbonyls (0.609 < *r* < 0.857) except for the cig-a-like with a grape flavored e-liquid (0.380 < *r* < 0.478) and the JUUL e-cigarette with all tested flavors (0.296 < *r* < 0.429) (Table 2).

Different conventional cigarettes were reported to have strong linear relationship (r^2^ = 0.90–0.95) between emitted CO and acetaldehyde [43]. This is because their formation is dominated by thermal decomposition and incomplete combustion of tobacco leaf and the wrapping paper, which does not differ significantly among different cigarettes [44]. Since the bulk of any e-liquid is composed of PG and VG, and if these components dominated CO and carbonyl production as was proposed in other studies [6,45], there would be no differences in CO-carbonyl relationship among different flavored e-liquids. However, we observed a wide variation in slopes and intercepts for the different e-cigarette devices and e-liquids tested in this study. This proves that CO and carbonyl emissions are dominated by e-liquid components other than PG and VG, such as flavoring compounds.

### 3.5. E-Cigarette vs. Conventional Cigarette Emissions

Carbonyl and CO emissions from the tested e-cigarettes were generally lower than those from conventional cigarettes except for formaldehyde emissions from the top-coil e-cigarette. According to the previous reports, conventional cigarettes yielded 81.5–187.7 μg of nicotine, 0.75–1.73 mg of CO, 1.08–15.4 μg of formaldehyde, and 5.88–81.5 μg of acetaldehyde per puff, assuming 13 puffs per cigarette [43,46,47,48]. All of the tested e-cigarettes under our experimental conditions generated 40 to 3618 times less CO than conventional cigarettes. The cig-a-like, ‘mod’, and JUUL devices produced less formaldehyde (3- to 120-fold) and acetaldehyde (3- to 3950-fold) than conventional cigarettes. The top-coil device generated 24 to 41 times less acetaldehyde, but formaldehyde emissions were comparable (within factors of 0.5 to 1.3) to those of conventional cigarettes. It should be noted that, given the cancer potency factors [49] of formaldehyde (1.3 × 10^−5^) and acetaldehyde (1.3 × 10^−6^), the lung cancer risk due to top-coil e-cigarette use is only 5 times lower than that of conventional cigarette use. This raises questions about effectiveness of top-coil devices for smoking harm reduction.

Nicotine and nicotine-normalized carbonyl and CO emissions for the four e-cigarettes are listed in Table 3. Such normalization might help to better understand e-cigarette users’ exposure, because most e-cigarette users are former cigarette smokers or dual users [50] who usually consume e-liquids until they inhale sufficient levels of nicotine [51]. Thus, low nicotine yield e-cigarette users could vape more frequently and, thus, increase exposure to harmful chemicals. Different e-liquid types and vaping topographies measured in this study were combined to estimate carbonyl and CO emissions per nicotine mass. Levels of nicotine, CO, and carbonyl emissions for the conventional cigarette smoke were obtained from literatures [46,47,48,52]. The cig-a-like and ‘mod’ e-cigarettes emitted similar levels of nicotine even though the ‘mod’ e-cigarette was used with e-liquid that had 6 times lower nicotine content than cig-a-like device. This is because the ‘mod’ produces significantly more aerosol due to its higher power output [53]. For the same reason, the top-coil e-cigarette generated 18 times less nicotine than the ‘mod’ device with the same e-liquid (3 mg/mL nicotine content). In our study, we used a low nicotine liquid, sold mostly for ‘mod’ e-cigarettes, to test the top-coil device. It is likely that actual top-coil e-cigarette users will be using a higher nicotine content liquid as top-coil devices produce less aerosol than ‘mod’ e-cigarettes. Even if we assume that the higher nicotine content liquids offered in the market (e.g., 18–24 mg/mL) are used, nicotine intake for top-coil e-cigarette users still would be 2.6 times lower than the ‘mod’ device users with lower nicotine content liquid (i.e., 3 mg/mL). The JUUL generated 2.9 times higher nicotine per puff than the conventional cigarette.

The tested e-cigarette devices usually shown to have lower nicotine-normalized carbonyls and CO emissions than conventional tobacco cigarettes. The cig-a-like, ‘mod’, and JUUL e-cigarettes showed lower nicotine-normalized CO (517- to 10,802-fold), formaldehyde (2.8- to 215-fold), acetaldehyde (17- to 9156-fold), and acrolein (78- to 6529-fold) emissions than those of conventional cigarettes. Assuming that an 18–24 mg/mL nicotine content is more realistic for top-coil e-cigarette than 3 mg/mL used in our study, we adjusted nicotine-normalized emissions by a factor of 7. Nicotine-normalized CO, acetaldehyde, and acrolein emissions for the top-coil device were then 20, 62, and 84 times lower than conventional cigarettes, respectively, while nicotine-normalized formaldehyde emissions were still 1.6 times higher. Cig-a-like, top-coil, and ‘mod’ devices generated 2–40 times more glyoxal per unit nicotine than conventional cigarettes. Glyoxal emissions could be of concern, as glyoxal was reported [54] to cause allergic reaction at 9.4–21 μg/m^3^, which is similar or lower than estimated daily glyoxal exposures for the cig-a-like, top-coil, and ‘mod’ e-cigarette users (assuming 200 puffs/day).

### 3.6. Public Health Implications

To our knowledge, this is the first study of carbonyl and CO emissions from JUUL devices under various vaping conditions. Carbonyl and CO emissions from the JUUL were significantly lower than those of conventional cigarettes and the other e-cigarettes tested in this study. JUUL is currently the most popular e-cigarette device in the U.S. [27,28]. Low carbonyl and CO emissions observed in our study and the nondetectable levels of benzene reported by [9] suggest that JUUL devices might benefit tobacco-use cessation efforts. However, it should be pointed out that the high nicotine content of the JUUL (59 mg/mL) could be a problem for the United States Food and Drug Administration (U.S. FDA)’s efforts to combat nicotine addiction [55]. Recently, the FDA proposed a new rule to lower the maximum allowable nicotine content of combustible cigarettes [55]. The JUUL e-cigarette generated 3 to 13 times more nicotine than the non-addictive nicotine level proposed by the FDA (0.5 mg/cig, 40–50 μg/puff) [55]. Potential harm caused by tobacco products is closely connected with the addictiveness of nicotine [56]. Indeed, young adults who used e-cigarettes were shown to be 4 times more likely to start conventional cigarette smoking than non-e-cigarette users [57]. Moreover, among adolescent and young adult e-cigarette users, pod-based e-cigarette users (e.g., JUUL) reported much higher frequency of vaping within 30 days (58.8%) than users of other e-cigarettes (30.1%) and conventional cigarettes (28.3%) [58]. Thus, the high nicotine addictiveness of JUUL could still harm public health by initiating new tobacco product users.

Moreover, it is worth mentioning that daily exposure estimates for the all tested e-cigarettes including JUUL could exceed acute exposure limits. Daily carbonyl exposures were estimated using measured emissions in this study, reported vaping frequency (200 puffs/day) [18,31], daily air inhalation volume (16 m^3^/day) [59] for healthy adult population. Estimated daily formaldehyde (83.8 μg/m^3^/day) and acetaldehyde (18.0 μg/m^3^/day) exposures for the top-coil e-cigarette were shown to exceed an acute minimal risk level (MRL) for 1–14 days average formaldehyde exposure (MRL = 50 μg/m^3^) [60] and the reference concentration (RfC = 9 μg/m^3^) [49] for daily average acetaldehyde exposure concentration, respectively. All tested e-cigarettes showed 9–104 times higher daily acrolein exposures than RfC value (0.02 μg/m^3^). The acute exposure estimates indicate that, despite e-cigarettes usually shown to generate less harmful chemicals than conventional cigarettes, e-cigarettes are not harmless. Thus, use of e-cigarettes, especially JUUL-like devices, among youth and non-smokers should be addressed to promote public health.

### 3.7. Limitations

This study had several limitations. First of all, even though we tested the four most popular e-cigarettes under a wide range of vaping conditions, we only included one e-cigarette in each device category due to limited resources available. As a recent study showed, a ‘mod’ e-cigarette with different coil configurations could generate varying levels of carbonyls under the same vaping pattern [53]. It is worth mentioning that extreme vaping conditions (e.g., ‘mod’ device with over 100-watt power output) could also alter carbonyls and CO emissions. Thus, the results of our study should be carefully examined to extrapolate beyond our experimental conditions. Further, the high amounts of PG/VG particles emitted from e-cigarettes (especially the ‘mod’ device) might affect carbonyl sampling efficiency. However, we used the best available carbonyl sampling method (DNPH filter followed by DNPH cartridge) for both particle and gas phase compounds, while other studies used a DNPH cartridge or an impinger containing DNPH solution, which is not suitable for samples containing large amounts of particles [61].

## 4. Conclusions

This study provides a comprehensive examination of how different e-cigarette types and use patterns affect e-cigarette emissions of carbonyls and CO. To the best of the authors’ knowledge, this is the first systematic study simultaneously measuring both carbonyl and CO levels emitted from different types of e-cigarettes. The study demonstrates that higher carbonyl and CO levels are associated with higher power settings, longer puff durations, and e-liquids with flavors. The top-coil type was found to produce the highest emissions among the studied devices under the vaping conditions used in this study. The ‘pod’ type device (i.e., JUUL) could emit significantly higher levels of nicotine than other devices, which could increase the risk of addiction, especially among the youth. Therefore, regulatory agencies should better inform youth and their parents to minimize the potential risks of vaping and public health burdens. Regulations of both e-liquid nicotine content (e.g., below non-addictive levels discussed in Section 3.5) and harmful chemical emissions should also be considered to better protect public health.

Carbonyl and CO emissions correlated with each other for all e-cigarette devices and e-liquids studied. However, these correlations varied among devices and e-liquid flavors, which suggests that chemical processes leading to carbonyl and CO formation depend largely on e-liquid components other than PG and VG, i.e., on e-liquid flavorings. These results have significant implications for regulating e-cigarette flavorings as they clearly influence production of harmful constituents. This study also demonstrated that carbonyl and CO emissions not only strongly depend on vaping topography, but that this dependence also varies among e-cigarette types. An important implication of this finding is that the current e-cigarette testing protocols, such as International Organization for Standardization (ISO) [62] and Cooperation Centre for Scientific Research Relative to Tobacco (CORESTA) [34] that use fixed puff topography (3-s puff duration, 55 mL puff volume, and 30-s interval), cannot adequately predict e-cigarette emissions at the wide range of real-world e-cigarette vaping patterns. A more robust e-cigarette testing protocol is needed to better aid regulators, policy makers, and public health professionals.

## Figures and Tables

**Table 1:** E-cigarette brands, e-liquid types, and e-cigarette aerosol generation conditions used in this study ^1^.

Type	E-liquid (Flavor, Nicotine)	Puff Duration (s)	Puff Volume (mL)	Power Output (watt)
Cig-a-like	Tobacco, 18 mg/mL	2, 3, 4, 5	100, 133	5.2
	Grape, 18 mg/mL	4	100	5.2
Top-coil	Strawberry-watermelon, 3 mg/mL	2, 3, 4, 5	40, 67, 100, 133	4.9
‘mod’	Strawberry-watermelon, 3 mg/mL	2, 3, 4, 5	40, 67, 100, 133	40, 50, 60
	Peach-lemonade, 3 mg/mL	4	40	50
	Vanilla-almond-milk, 6 mg/mL	4	40	50
	Unflavored, no-nicotine	4	40	40, 50, 60
‘pod’ (JUUL)	Fruit melody, 59 mg/mL	2, 3, 4, 5	100, 133	6–9
	Cool mint, 59 mg/mL	4	100	6–9
	Virginia tobacco, 59 mg/mL	4	100	6–9
	Creme brulee, 59 mg/mL	4	100	6–9

^1^ The effect of different puff durations (2, 3, 4, and 5 s) and puff volumes (40, 67, 100, and 133 mL) were tested at a fixed flow rate (1.5 L/min) and puff duration (4 s), respectively. Power output conditions for the Brand III e-cigarette were tested under 40 mL puff volume and 4-s puff duration.

**Table 2:** Correlation coefficients of CO and carbonyl compounds in e-cigarette aerosols. Asterisk marks (*) indicate Pearson’s correlation coefficients with statistical significance (*p* < 0.05).

E-Cigarette Type	Formaldehyde	Acetaldehyde	Acrolein	Glyoxal
Cig-a-like (tobacco)	0.820 *	0.821 *	0.837 *	0.823 *
Cig-a-like (grape)	0.476	0.380	–	0.478
Top-coil	0.909 *	0.857 *	0.901 *	0.620 *
‘mod’	0.847 *	0.855 *	0.776 *	0.609 *
JUUL	0.335	0.429	–	0.296

**Table 3:** Nicotine (μg/puff) and nicotine-normalized CO (CO_n_), formaldehyde (formaldehyde_n_), acetaldehyde (acetaldehyde_n_), acrolein (acrolein_n_), and glyoxal (glyoxal_n_) concentrations (ng/puff/μg nicotine) for four tested e-cigarettes. Values for conventional cigarettes are given for comparison ^1^.

Device Type	Nicotine (μg/puff)	Nicotine Normalized Concentration (ng/puff/μg Nicotine)				
		CO_n_	Formaldehyde_n_	Acetaldehyde_n_	Acrolein_n_	Glyoxal_n_
Cig-a-like	104 ± 10.4	13.8 ± 68.8	25.6 ± 6.13	31.6 ± 16.4	0.47 ± 1.7	13.8 ± 68.8
Top-coil ^2^	6.0 ± 1.3	556 ± 337	114 ± 427	8.92 ± 38.5	1.52 ± 6.99	556 ± 337
‘mod’	106 ± 11.8	21.1 ± 76.3	7.77 ± 1.12	2.61 ± 0.54	1.64 ± 2.89	21.1 ± 76.3
JUUL	390 ± 30.5	1.01 ± 7.14	0.34 ± 4.34	0.06 ± 2.06	0.02 ± 0.66	1.01 ± 7.14
Cigarette ^3^	134 ± 75.1	10893 ± 7313	72.4 ± 84.8	551 ± 669	127 ± 78.4	10893 ± 7313

^1^ Nicotine, CO, and carbonyl data from the two (tobacco and fruit [1.8% nicotine]), one (fruit [0.3% nicotine]), one (fruit [0.3% nicotine]), and four (fruit, mint, tobacco, and cream [5% nicotine]) e-liquids were combined for the cig-a-like, top-coil, ‘mod’, and JUUL e-cigarettes under 67–133 mL puff volumes and 3–5-s puff durations, respectively. ^2^ Nicotine-normalized carbonyl and CO emissions were adjusted by 7 times to reflect top-coil users’ e-liquid nicotine contents (see explanation in the text). ^3^ Nicotine, CO, and carbonyl emissions for conventional cigarette were obtained from [46,47,48,52].

## Supplementary Materials

The following are available online at https://www.mdpi.com/1660-4601/17/8/2767/s1. Figure S1: E-cigarette devices used in this study. Figure S2: Scheme of E-cigarette aerosol testing system. Figure S3: Example signal obtained from the CO analyzer. Table S1: Descriptions of the e-cigarette brands, e-liquid types, and e-cigarette aerosol generation conditions used in this study. Table S2: Detailed descriptions of chromatographic condition for the carbonyl-DNPH analysis. Table S3: Limit of detections (LOD) and limit of quantifications (LOQ) for carbonyl compounds. Table S4: Detailed descriptions of chromatographic condition for the nicotine analysis. Table S5: Limit of detections (LOD) and limit of quantifications (LOQ) for carbonyl compounds. Table S6: Carbonyl levels (ng/puff) and e-liquid consumption (mg/puff) under different puff durations. Blank values indicate below LOD or LOQ. Table S7: Linear regression parameters (estimate ± standard error) for e-cigarette emissions and puff durations (emission = β1 × duration + β0). Table S8: Nonlinear regression parameters (estimate ± standard error) for top-coil emissions and puff durations (emission = a × e^(b × duration)^). Table S9: Carbonyl levels (ng/puff) and e-liquid consumption (mg/puff) under different puff flow rates. Blank values indicate below LOD or LOQ. Cig-a-like and JUUL were not tested under 10 and 17 mL/s puff flow rates. Table S10: Linear regression parameters (estimate ± standard error) between e-cigarette emissions and flow rates (emission = β1 × flow rate + β0).
