# Supplementary material for: Carbonyls and Carbon Monoxide Emissions from Electronic Cigarettes Affected by Device Type and Use Patterns
Source: Int J Environ Res Public Health. 2020 Apr 17;17(8):2767. doi: 10.3390/ijerph17082767 (PMC7215697; doi:10.3390/ijerph17082767)
Supplement: Supplementary file 1 [file ijerph-17-02767-s001.pdf]

Supplementary Materials

I. Tested electronic cigarette devices, e-liquids, and puffing conditions

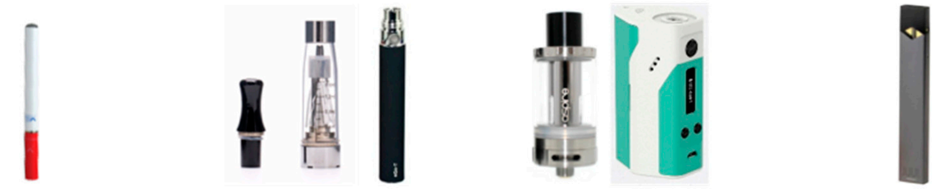

(a) Cig-a-like (b) Top-coil (c) 'mod' (d) 'pod'

Figure S1. E-cigarette devices used in this study.

Table S1. Descriptions of the e-cigarette brands, e-liquid types, and e-cigarette aerosol generation conditions used in this study.

E-cigarette type		E-liquid		Puff duration (seconds)	Puff volume (ml)	Power Output (watt)
Cig-a-like:	V2 Standard (single coil with cotton fiber, V2 standard battery)	Type A:	V2 RED tobacco (PG, 18 mg/ml nicotine, water, with tobacco flavor)	2, 3, 4, 5	100, 133	5.2
		Type B:	V2 Grape (PG, 18 mg/ml nicotine, water, with grape flavor)	4	100	5.2
Top-coil:	eGo CE4 (single-top coil with cotton wick, eGo-T battery)	Type C:	Starstruk Watermelon (6:4 = VG:PG, 3 mg/ml nicotine, strawberry-watermelon flavor)	2, 3, 4, 5	40, 67, 100, 133	4.9
'mod':	Aspire Cleito (clapton style Kanthal coil in a modified chimney with cotton wick, Reuleaux RX200 mods)	Type C:	Starstruk Watermelon (6:4 = VG:PG, 3 mg/ml nicotine, strawberry-watermelon flavor)	2, 3, 4, 5	40, 67, 100, 133	40, 50, 60
		Type D:	Sweet LEAF (8:2 = VG:PG, 3 mg/ml nicotine, peach-sweet tea lemonade flavor)	4	40	50
		Type E:	MOO (7:3 = VG:PG, 6 mg/ml nicotine, vanilla-almond-milk flavor)	4	40	50
		Type F:	Non-flavored (7:3 = VG:PG, non-flavored)	4	40	40, 50, 60
'pod':	JUUL (Nichrome coil with silica wick, lithium-Ion Polymer battery with a temperature control feature)	Type G:	Fruit melody (VG/PG [unknown ratio], 59 mg/ml nicotine-salt, benzoic acid, fruit flavor)	2, 3, 4, 5	100, 133	6–9
		Type H:	Cool mint (VG/PG [unknown ratio], 59 mg/ml nicotine-salt, benzoic acid, mint flavor)	4	100	6–9
		Type I:	Virginia tobacco (VG/PG [unknown ratio], 59 mg/ml nicotine-salt, benzoic acid, tobacco flavor)	4	100	6–9
		Type J:	Creme brulee (VG/PG [unknown ratio], 59 mg/ml nicotine-salt, benzoic acid, cream flavor)	4	100	6–9

Note: Puff durations and puff volumes were tested at fixed flow rate (1.5 L/min) and puff duration (4-sec), respectively. Power output conditions for the Brand III e-cigarette were tested under 40 ml puff volume and 4-sec puff duration.

II. Experimental Setting

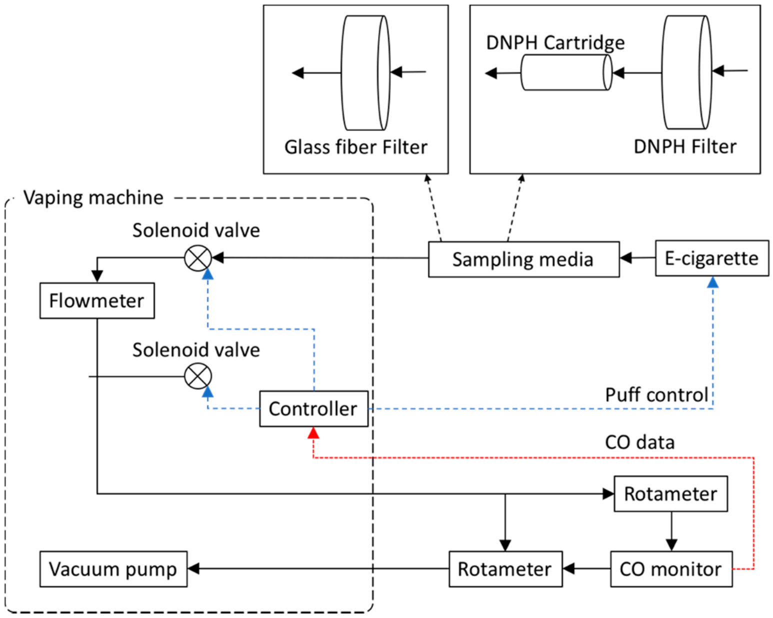

Figure S2. Scheme of E-cigarette Aerosol Testing System.

III. CO measurements

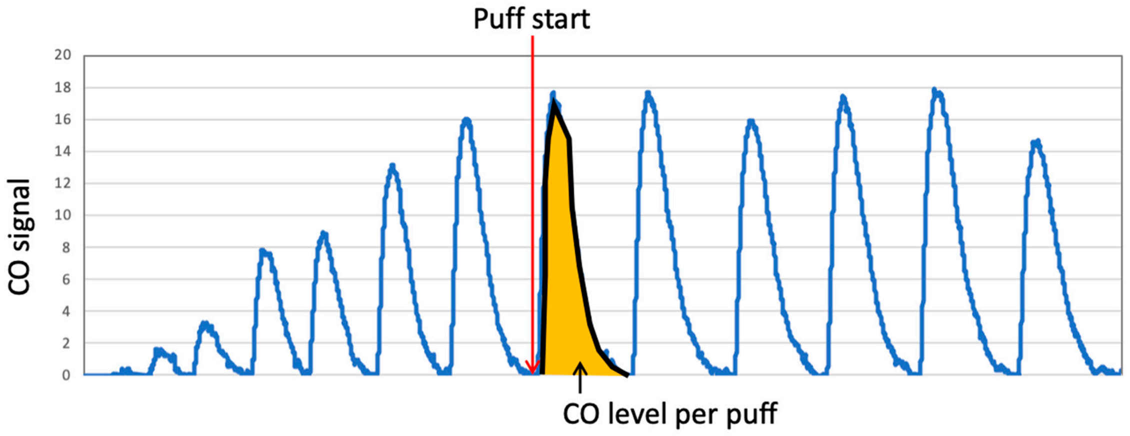

Figure S3. Example signal obtained from the CO analyzer.

Concentrations of CO emitted from the e-cigarettes were measured using a Model 8830 CO Analyzer (Teledyne Monitor Labs, Englewood, CO, USA) connected to the vaping machine via a 2 m long 1/4" outer-diameter Teflon tubing (Figure S1). Sample flow rate was 0.6 L/min which was equal or lower than puff flow rates (0.6–2.0 L/min). The flow rates were controlled by two rotameters and a flow controller attached on the CO analyzer. A quartz fiber filter was installed at the inlet of the CO analyzer to protect the instrument. The analog output of the analyzer (Figure S3) was continuously recorded at a rate of 5 Hz (a measurement per 0.2-sec) using a digital-analog converter (LabJack U6 series, LabJack Corporation, Lakewood, CO, USA). The total amount of CO produced per puff was calculated by integrating peaks in CO readings and multiplying them by the instrument sample flow rate as shown in Figure S3. The CO analyzer signals shown to decrease to background level within the puff interval. No interference from PG or VG in CO measurements was detected.

IV. Detailed Analytical Method for Carbonyl-DNPH Species

Table S2. Detailed descriptions of chromatographic condition for the carbonyl-DNPH analysis.

Parameter	Description
Instrument:	Waters 2690 Alliance System with a model 996 photodiode array detector (DAD)
Column:	Agilent Polaris 3 column, C18-A, 3 μm, 100 × 2.0 mm
Mobile phase:	Ultrapure water (Phase A), Acetonitrile (ACN, Phase B)
Gradient:	At 0 min 42% ACN<br>At 9 min 42% ACN<br>At 16 min 55% ACN<br>At 18 min 55% ACN<br>At 19 min 90% ACN<br>At 25 min 90% ACN<br>At 26 min 42% ACN<br>At 30 min 42% ACN
Flow rate:	0.25 ml/min
DAD:	360 nm
Injection volume:	2 μl

Table S3. Limit of detections (LOD) and limit of quantifications (LOQ) for carbonyl compounds.

	Retention time (min)	LOD (μg/ml)	LOQ (μg/ml)
Formaldehyde	4.57	0.011	0.037
Acetaldehyde	6.42	0.022	0.074
Acetone	9.25	0.016	0.054
Acrolein	10.0	0.015	0.052
Propionaldehyde	11.3	0.018	0.061
Crotonaldehyde	15.6	0.078	0.259
2-Butanone (MEK)	16.4	0.028	0.093
Methacrolein	17.0	0.023	0.078
n-Butyraldehyde	17.4	0.019	0.062
Benzaldehyde	20.2	0.016	0.052
Valeraldehyde	21.8	0.017	0.056
Glyoxal	22.4	0.170	0.565
m-tolualdehyde	23.0	0.085	0.285
Hexaldehyde	23.6	0.019	0.062

V. Detailed Analytical Method for Nicotine

Table S4. Detailed descriptions of chromatographic condition for the nicotine analysis.

Parameter	Description
Instrument:	Waters 2690 Alliance System with a model 996 photodiode array detector (DAD)
Column:	Agilent Polaris 3 column, C18-A, 3 μm, 100 × 2.0 mm
Mobile phase:	Phosphate buffer (pH 7.9, 8.5 mM Na_2HPO_4 [Electron Microscopy Sciences, PA, USA], 1.5 mM KH_2PO_4 [Beantown Chemical Corporation, NH, USA], Phase A), Methanol (MeOH, Phase B)
Gradient:	At 0 min 30% MeOH<br>At 2.5 min 30% MeOH<br>At 6 min 5% MeOH<br>At 8 min 70% MeOH<br>At 12 min 70% MeOH<br>At 15 min 5% MeOH<br>At 20 min 5% MeOH<br>At 25 min 30% MeOH<br>At 30 min 30% MeOH
Flow rate:	0.1 ml/min
DAD:	260 nm for nicotine, 220 nm for quinoline
Injection volume:	1 μl

Table S5. Limit of detections (LOD) and limit of quantifications (LOQ) for carbonyl compounds.

	Retention time (min)	LOD (μg/ml)	LOQ (μg/ml)
Nicotine	21.2	0.44	1.47
Quinoline	22.4	0.90	2.99

VI. Carbonyl, CO Emissions under Different Puff Topographies and Regression Analysis Results

Table S6. Carbonyl levels (ng/puff) and e-liquid consumption (mg/puff) under different puff durations. Blank values indicate below LOD or LOQ.

	Cig-a-like				Top-coil				'mod'				JUUL			
Flow (ml/sec)	25	25	25	25	25	25	25	25	25	25	25	25	25	25	25	25
Duration (Sec)	2	3	4	5	2	3	4	5	2	3	4	5	2	3	4	5
E-liquid	3.9 ± 0.1	4.1 ± 0.1	4.5 ± 0.3	5.2 ± 0.4	3.5 ± 0.4	4.2 ± 0.2	4.8 ± 0.2	5.1 ± 0.4	18.1 ± 3.9	30.7 ± 7.1	47.5 ± 10.9	87.7 ± 10.1	2.0 ± 0.2	2.1 ± 0.2	2.8 ± 0.2	3.3 ± 0.3
CO	0.59 ± 0.10	0.72 ± 0.14	1.42 ± 0.71	2.2 ± 0.63	9.06 ± 2.09	15.4 ± 3.12	23.3 ± 3.07	36.2 ± 10.0	0.72 ± 0.18	1.6 ± 0.53	2.24 ± 0.90	2.74 ± 0.56	0.21 ± 0.05	0.17 ± 0.05	0.27 ± 0.07	0.27 ± 0.05
Formaldehyde	0.53 ± 0.04	1.35 ± 0.17	2.65 ± 0.06	3.91 ± 0.23	0.5 ± 0.24	1.31 ± 0.51	4.80 ± 3.88	16.9 ± 9.40	0.09 ± 0.01	0.21 ± 0.04	0.47 ± 0.01	0.57 ± 0.16	0.03 ± 0.01	0.04 ± 0.01	0.14 ± 0.04	0.18 ± 0.12
Acetaldehyde	0.73 ± 0.06	1.74 ± 0.23	3.27 ± 0.17	4.15 ± 0.18	0.14 ± 0.05	0.27 ± 0.13	0.56 ± 0.19	4.44 ± 3.33		0.03 ± 0.01	0.08 ± 0.01	0.1 ± 0.02		0.01 ± 0.01	0.01 ± 0.01	0.02 ± 0.01
Acetone	0.24 ± 0.01	0.33 ± 0.02	0.45 ± 0.04	0.56 ± 0.01	0.11 ± 0.01	0.10 ± 0.01	0.12 ± 0.02	0.11 ± 0.09					0.12 ± 0.01	0.11 ± 0.01	0.11 ± 0.01	0.12 ± 0.01
Acerolein	0.03 ± 0.01	0.03 ± 0.01	0.05 ± 0.02	0.08 ± 0.01			0.06 ± 0.06	0.25 ± 0.14	0.01 ± 0.02	0.06 ± 0.04	0.17 ± 0.03	0.23 ± 0.01				
Propionaldehyde	0.11±0.01	0.28±0.04	0.59±0.08	0.86±0.04	0.01±0.02		0.06±0.06	0.42±0.11								
Crotonaldehyde			0.34 ± 0.57	0.64 ± 0.55		0.01 ± 0.02							0.01 ± 0.02			0.01 ± 0.02
2-Butanone	1.86 ± 0.03	2.20 ± 0.13	1.79 ± 0.61	1.66 ± 0.38		0.42 ± 0.65		0.2 ± 0.31					1.17 ± 0.02	1.19 ± 0.01	1.13 ± 0.03	1.06 ± 0.06
Methacrolein			0.20±0.17	0.12±0.21			0.16 ± 0.27	0.23 ± 0.1								
n-Butyraldehyde	0.01 ± 0.02		0.01 ± 0.02		0.01 ± 0.02	0.03 ± 0.02	0.06 ± 0.07	0.33 ± 0.31								
Bezaldehyde	0.01 ± 0.01	0.02 ± 0.02	0.02 ± 0.02	0.03 ± 0.01	0.06 ± 0.01	0.09 ± 0.01	0.11 ± 0.01	0.14 ± 0.02	0.49 ± 0.04	0.88 ± 0.1	1.33 ± 0.05	1.59 ± 0.05	0.01 ± 0.02	0.03 ± 0.01	0.03 ± 0.01	0.03 ± 0.01
Valeraldehyde	0.02 ± 0.01	0.04 ± 0.01	0.06 ± 0.01	0.13 ± 0.02	0.09 ± 0.03	0.11 ± 0.03	0.41 ± 0.19	0.43 ± 0.22			0.02 ± 0.01	0.03 ± 0.02		0.02 ± 0.01	0.04 ± 0.01	0.05 ± 0.03
Glyoxal	0.34 ± 0.07	0.73 ± 0.11	1.35 ± 0.17	2.99 ± 0.72	1.16 ± 1.03	2.72 ± 2.36	5.01 ± 1.65	7.04 ± 3.19	0.18 ± 0.03	0.32 ± 0.11	0.67 ± 0.06	0.69 ± 0.16	0.01 ± 0.02	0.05 ± 0.01	0.17 ± 0.06	0.24 ± 0.19
m-Tolualdehyde	0.07 ± 0.02	0.14 ± 0.02	0.18 ± 0.11	0.28 ± 0.14	0.09 ± 0.08	0.22 ± 0.11	0.54 ± 0.12	0.55 ± 0.11		0.02 ± 0.01	0.06 ± 0.01	0.07 ± 0.02	0.02 ± 0.01	0.04 ± 0.01	0.06 ± 0.01	0.07 ± 0.01
Hexaldehyde	0.28 ± 0.03	0.65 ± 0.05	1.20 ± 0.10	1.83 ± 0.25	0.40 ± 0.13	0.53 ± 0.15	0.91 ± 0.13	0.63 ± 0.14	0.29 ± 0.02	0.38 ± 0.35	1.09 ± 0.02	1.08 ± 0.18	0.06 ± 0.01	0.13 ± 0.01	0.20 ± 0.01	0.24 ± 0.04

Table S7. Linear regression parameters (estimate±standard error) for e-cigarette emissions and puff durations (emission = β1 x duration + β0).

Type	Formaldehyde		Acetaldehyde		Acrolein		Glyoxal		CO	
	Slope (β1)	Intercept (β0)	Slope (β1)	Intercept (β0)	Slope (β1)	Intercept (β0)	Slope (β1)	Intercept (β0)	Slope (β1)	Intercept (β0)
Cig-a-like[*]	1.14 ± 0.049	−1.89 ± 0.18	1.18 ± 0.055	−1.66 ± 0.20	0.018 ± 0.0027	−0.014 ± 0.0099	0.86 ± 0.00005	−1.64 ± 0.0054	0.55 ± 0.12	−0.69 ± 0.45
	**p < 0.001**[***]		**p < 0.001**[***]		**p < 0.001**[***]		**p < 0.001**[***]		**p < 0.001**[***]	
Top-coil[*]	5.27 ± 1.43	−12.6 ± 5.27	1.30 ± 0.49	−3.24 ± 1.81	0.080 ± 0.022	−0.203 ± 0.083	1.99 ± 0.51	−2.99 ± 1.88	8.92 ± 1.37	−10.2 ± 5.04
	**p = 0.004**[***]		**p = 0.025**[***]		**p = 0.005**[***]		**p = 0.003**[***]		**p < 0.001**[***]	
	Residual = 5.55		Residual = 1.91		Residual = 0.09		Residual = 1.99		Residual = 5.31	
'mod'	0.17 ± 0.021	−0.26 ± 0.078	0.035 ± 0.0035	−0.070 ± 0.013	0.077 ± 0.0075	−0.15 ± 0.028	0.19 ± 0.030	−0.19 ± 0.11	0.67 ± 0.14	−0.52 ± 0.52
	**p < 0.001**[***]		**p < 0.001**[***]		**p < 0.001**[***]		**p < 0.001**[***]		**p < 0.001**[***]	
JUUL	0.054 ± 0.015	−0.088 ± 0.055	0.0050 ± 0.0009	−0.0078 ± 0.0035	NA[**]		0.081 ± 0.024	−0.17 ± 0.087	0.028 ± 0.015	0.14 ± 0.056
	**p = 0.005**[***]		**p < 0.001**[***]				**p = 0.007**[***]		p = 0.100	

[*]Regression analysis for acrolein from cig-a-like and top-coil ECIG was done using 4 and 5-sec; [**]Non-applicable due to limited data; [***]Significant relationships (p<0.05) are indicated in bold

Table S8. Non-linear regression parameters (estimate±standard error) for top-coil emissions and puff durations [emission = a x $e^{(b \times duration)}$].

Type	Formaldehyde		Acetaldehyde		Acrolein		Glyoxal		CO	
	a	b	a	b	a	b	a	b	a	b
Top-coil	0.031 ± 0.075	1.26 ± 0.48	0.00030 ± 0.0024	1.92 ± 1.62	0.000010 ± 0.000037	1.56 ± 0.72	0.63 ± 0.46	0.49 ± 0.16	3.96 ± 1.32	0.44 ± 0.074
	p* = 0.027		*p* = 0.266		*p* = 0.055		***p* = 0.013***		***p* < 0.001***	
	Residual = 4.56		Residual = 1.58		Residual = 0.07		Residual = 2.02		Residual = 5.00	

*Significant relationships (p<0.05) are indicated in bold

Table S9. Carbonyl levels (ng/puff) and e-liquid consumption (mg/puff) under different puff flow rates. Blank values indicate below LOD or LOQ. Cig-a-like and JUUL was not tested under 10 and 17 ml/sec puff flow rates.

	Cig-a-like		Top-coil				mod'				JUUL	
Flow (ml/sec)	25	33	10	17	25	33	10	17	25	33	25	33
Duration (Sec)	4	4	4	4	4	4	4	4	4	4	4	4
E-liquid	4.5 ± 0.3	5.0 ± 0.2	4.2 ± 1.1	4.5 ± 0.4	4.8 ± 0.2	5.3 ± 0.1	18.3 ± 1.2	39.3 ± 1.5	47.5 ± 10.8	84.0 ± 29.6	2.8 ± 0.2	2.8 ± 0.2
CO	1.42 ± 0.71	2.60 ± 0.20	29.9 ± 6.70	26.9 ± 5.35	23.3 ± 3.07	19.1 ± 1.59	2.40 ± 0.81	1.95 ± 0.97	2.24 ± 0.90	3.78 ± 0.87	0.27 ± 0.07	0.37 ± 0.12
Formaldehyde	2.65 ± 0.06	2.93 ± 0.27	16.9 ± 0.55	8.83 ± 3.61	4.80 ± 3.88	6.13 ± 1.37	0.41 ± 0.10	0.42 ± 0.04	0.47 ± 0.01	0.83 ± 0.16	0.14 ± 0.04	0.07 ± 0.07
Acetaldehyde	3.27 ± 0.17	3.55 ± 0.42	1.85 ± 1.76	1.62 ± 1.56	0.37 ± 0.35	1.08 ± 0.15	0.14 ± 0.07	0.06 ± 0.01	0.08 ± 0.01	0.28 ± 0.06	0.01 ± 0.01	0.01 ± 0.02
Acetone	0.45 ± 0.04	0.09 ± 0.01	0.21 ± 0.04	0.15 ± 0.02	0.12 ± 0.02	0.09 ± 0.01	0.03 ± 0.02			0.07 ± 0.01	0.11 ± 0.01	
Acerolein	0.05 ± 0.02		0.44 ± 0.03	0.13 ± 0.07	0.06 ± 0.06	0.14 ± 0.02	0.05 ± 0.02	0.16 ± 0.02	0.17 ± 0.03	0.13 ± 0.06		
Propionaldehyde	0.59 ± 0.08	0.09 ± 0.02	0.12 ± 0.08	0.12 ± 0.07	0.06 ± 0.06	0.05 ± 0.01	0.02 ± 0.01			0.03 ± 0.01		
Crotonaldehyde	0.34 ± 0.57			0.06 ± 0.11						0.01 ± 0.01		0.01 ± 0.01
2-Butanone	1.79 ± 0.61		0.02 ± 0.01			0.01 ± 0.00	0.07 ± 0.01			0.22 ± 0.10	1.13 ± 0.03	
Methacrolein	0.20 ± 0.17	0.04 ± 0.01		0.25 ± 0.23	0.16 ± 0.27	0.20 ± 0.01	0.12 ± 0.05	0.04 ± 0.04		0.22 ± 0.04		
n-Butyraldehyde	0.01 ± 0.02		0.27 ± 0.06	0.08 ± 0.07	0.06 ± 0.07							
Bezaldehyde	0.02 ± 0.02		0.03 ± 0.01	0.14 ± 0.02	0.11 ± 0.01	0.05 ± 0.00	0.19 ± 0.02	1.47 ± 0.03	1.33 ± 0.05	0.42 ± 0.17	0.03 ± 0.01	0.02 ± 0.02
Valeraldehyde	0.06 ± 0.01		0.70 ± 0.15	0.54 ± 0.21	0.41 ± 0.19	0.39 ± 0.05	0.03 ± 0.01	0.03 ± 0.01	0.02 ± 0.01	0.04 ± 0.01	0.04 ± 0.01	
Glyoxal	1.35 ± 0.17	2.82 ± 0.31	5.86 ± 3.26	7.27 ± 0.24	5.01 ± 1.65	13.2 ± 3.62	0.2 ± 0.03	0.83 ± 0.08	0.67 ± 0.06	0.34 ± 0.11	0.17 ± 0.06	0.19 ± 0.19
m-Tolualdehyde	0.18 ± 0.11		0.18 ± 0.03	0.47 ± 0.4	0.54 ± 0.12	0.19 ± 0.15	0.08 ± 0.02	0.05 ± 0.01	0.06 ± 0.01	0.16 ± 0.06	0.06 ± 0.01	
Hexaldehyde	1.20 ± 0.10	1.98 ± 0.13	1.23 ± 0.64	1.70 ± 2.16	0.91 ± 0.13	2.30 ± 0.17	0.73 ± 0.08	0.91 ± 0.08	1.09 ± 0.02	0.96 ± 0.19	0.20 ± 0.01	0.15 ± 0.06

Table S10. Linear regression parameters (estimate±standard error) between e-cigarette emissions and flow rates (emission = β1 x flow rate + β0).

Type	Formaldehyde		Acetaldehyde		Acrolein		Glyoxal		CO	
	Slope (β1)	Intercept (β0)	Slope (β1)	Intercept (β0)	Slope (β1)	Intercept (β0)	Slope (β1)	Intercept (β0)	Slope (β1)	Intercept (β0)
Cig-a-like*	0.035 + 0.020<br>*p* = 0.148	1.76 + 0.58	0.035 + 0.03<br>*p* = 0.340	2.39 + 0.95	NA**		0.18+0.026<br>***p*=0.002*****	−3.26 + 0.75	0.15 + 0.054<br>*p* = 0.052	−2.26 + 1.57
Top-coil	−0.46 + 0.15<br>***p* = 0.010*****	19.0 + 3.33	−0.046 + 0.039<br>*p* = 0.266	2.20 + 0.89	−0.012 + 0.0041<br>***p* = 0.014*****	0.45 + 0.093	0.26+0.11<br>***p*=0.047*****	2.35 + 2.61	−0.47 + 0.14<br>***p* = 0.007*****	34.77 + 3.18
'mod'	0.017 + 0.0043<br>***p* = 0.003*****	0.17 + 0.099	0.0059 + 0.0029<br>*p* = 0.064	0.013 + 0.065	0.003 + 0.0019<br>*p* = 0.135	0.064 + 0.043	0.0025+0.009<br>*p*=0.799	0.46 + 0.22	0.059 + 0.032<br>*p* = 0.095	1.34 + 0.73
JUUL*	−0.008 + 0.0054<br>*p* = 0.215	0.34 + 0.16	−0.00023 + 0.001<br>*p* = 0.843	0.019 + 0.032	NA**		0.0026+0.015<br>*p*=0.869	0.10 + 0.43	0.013 + 0.010<br>*p* = 0.276	−0.046 + 0.30

*Regression analysis for cig-a-like and JUUL was done using two flow rates (25 and 33 ml/sec); **Non-applicable due to limited data; ***Significant relationships ($p<0.05$) are indicated in bold
